# The genome sequence of a druid fly, *Clusia tigrina *(Fallén, 1820)

**DOI:** 10.12688/wellcomeopenres.20109.1

**Published:** 2023-10-06

**Authors:** Liam M. Crowley, Ruth Y. Akinmusola, University of Oxford and Wytham Woods Genome Acquisition Lab, Darwin Tree of Life Barcoding collective, Wellcome Sanger Institute Tree of Life programme, Wellcome Sanger Institute Scientific Operations: DNA Pipelines collective, Tree of Life Core Informatics collective, Darwin Tree of Life Consortium

**Affiliations:** 1 Department of Biology, University of Oxford, Oxford, England, UK; 2 Department of Life Sciences, University of Bath, Bath, England, UK

**Keywords:** Clusia tigrina, a druid fly, genome sequence, chromosomal, Diptera

## Abstract

We present a genome assembly from an individual male *Clusia tigrina* (a druid fly; Arthropoda; Insecta; Diptera; Clusiidae). The genome sequence is 1,216.4 megabases in span. Most of the assembly is scaffolded into 5 chromosomal pseudomolecules, including the X and Y sex chromosomes. The mitochondrial genome has also been assembled and is 17.68 kilobases in length.

## Species taxonomy

Eukaryota; Metazoa; Eumetazoa; Bilateria; Protostomia; Ecdysozoa; Panarthropoda; Arthropoda; Mandibulata; Pancrustacea; Hexapoda; Insecta; Dicondylia; Pterygota; Neoptera; Endopterygota; Diptera; Brachycera; Muscomorpha; Eremoneura; Cyclorrhapha; Schizophora; Acalyptratae; Opomyzoidea; Clusiidae; *Clusia*; *Clusia tigrina* (Fallén, 1820) (NCBI:txid576317).

## Background

*Clusia tigrina* is a member of the Clusiidae family, subfamily Clusiinae (Halliday, 1838), commonly known as druid flies ( Lonsdale, 2017). Druid flies have characteristic antennae, in which the second segment has a triangular projection over the third segment when viewed laterally. Flies of this family have dark brown to pale yellow narrow bodies (2.5–6.0 mm long) and variable anterodistal wing infuscations ( Fu *et al.*, 2010; Kazerani *et al.*, 2020). Their species-specific morphologies include different patterns of spots and brown stripes ( Lonsdale, 2017). *C. tigrina* can be distinguished from other closely related druid flies, such as *C. flava*, by the three prominent dark brown marks on their wings ( Kazerani *et al.*, 2020).

Clusiidae are distributed worldwide, but most occur in tropical regions and only 15 species have been identified from Europe ( Hellqvist, 2018). They are more abundant in tropical regions, but it remains likely that more species await discovery in temperate biomes ( Lonsdale, 2017). *Clusia tigrina* has been recorded mainly in western Europe and Scandinavia, with sparse records from Serbia and Russia ( GBIF Secretariat, 2022). It is associated with forested habitats, with plenty of large, mature trees, since their saproxylic larvae develop in deadwood ( Roháček *et al*., 2017). *C. tigrina* is a rare fly in Britain and Ireland ( Falk, 1991), although recently there has been an increase in records.

Male *C. tigrina* (and other Clusiidae) have been observed in competitive courtship displays called ‘lekking’, in which they gather in one place for the purpose of attracting females to the area ( Rathore *et al.*, 2023).

Studies on the phylogeny and evolution of druid flies tend to rely on morphological data ( Lonsdale, 2017), which may be limited, therefore, integrating molecular data may provide a more comprehensive understanding. The availability of high-quality genome data could help reconstruct the phylogeny and evolutionary history of *C. tigrina*. Here, we present a chromosomally complete genome sequence for *C. tigrina* based on one male specimen from Wytham Woods, Oxfordshire. This is the first whole genome sequence for a member of Clusiidae, and it is anticipated that it will provide a foundation for understanding biodiversity, evolutionary history and the genetic variation underlying the different morphological traits of this group.

## Genome sequence report

**Figure 1.**
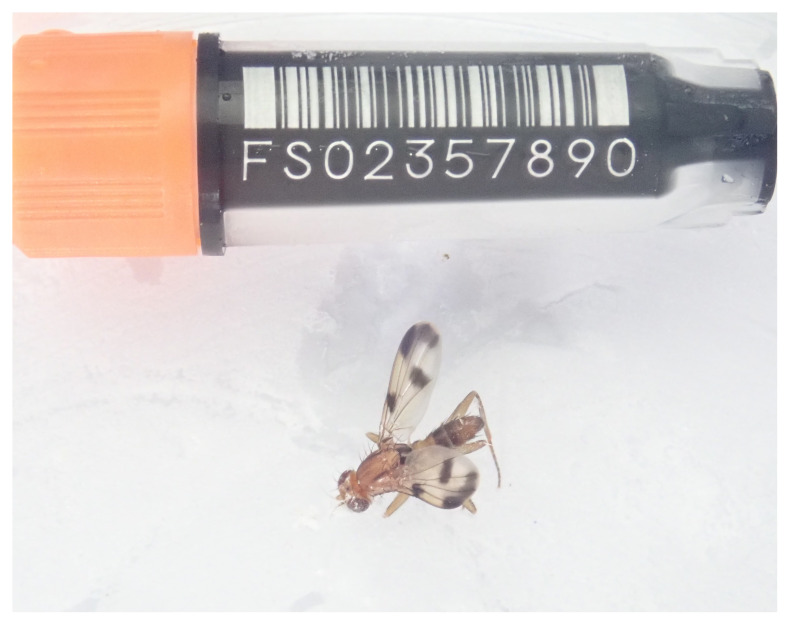
Photograph of the *Clusia tigrina* (idCluTigr1) specimen used for genome sequencing.

The genome was sequenced from one male *Clusia tigrina* ( Figure 1) collected from Wytham Woods, Oxfordshire, UK (latitude 51.76, longitude –1.32). A total of 31-fold coverage in Pacific Biosciences single-molecule HiFi long reads and 30-fold coverage in 10X Genomics read clouds was generated. Primary assembly contigs were scaffolded with chromosome conformation Hi-C data. Manual assembly curation corrected 271 missing joins or mis-joins and removed 5 haplotypic duplications, reducing the assembly length by 0.17% and the scaffold number by 24.06%, and increasing the scaffold N50 by 464.14%.

**Figure 2.**
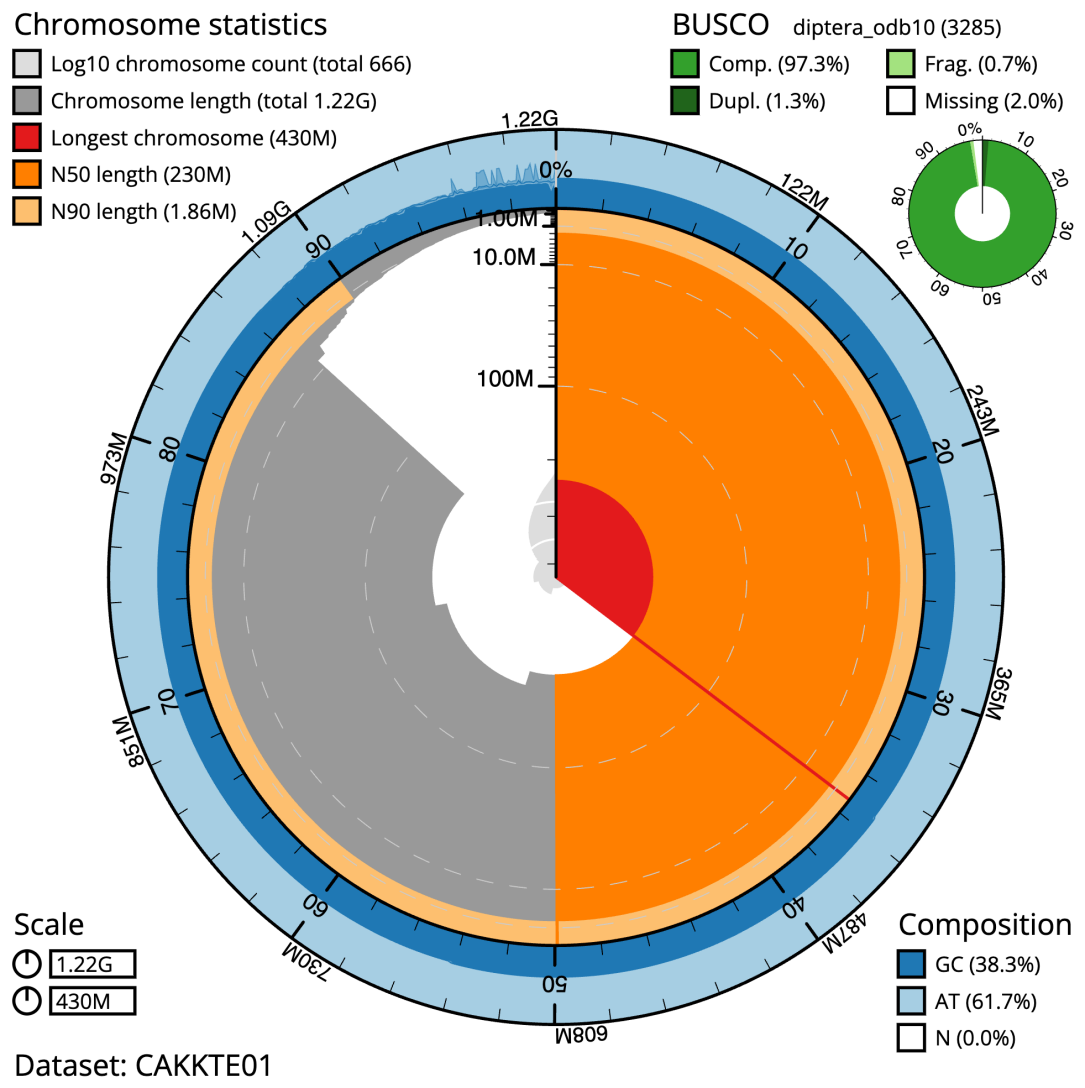
Genome assembly of *Clusia tigrina*, idCluTigr1.2: metrics. The BlobToolKit Snailplot shows N50 metrics and BUSCO gene completeness. The main plot is divided into 1,000 size-ordered bins around the circumference with each bin representing 0.1% of the 1,216,395,172 bp assembly. The distribution of scaffold lengths is shown in dark grey with the plot radius scaled to the longest scaffold present in the assembly (429,819,325 bp, shown in red). Orange and pale-orange arcs show the N50 and N90 scaffold lengths (230,177,572 and 1,864,154 bp), respectively. The pale grey spiral shows the cumulative scaffold count on a log scale with white scale lines showing successive orders of magnitude. The blue and pale-blue area around the outside of the plot shows the distribution of GC, AT and N percentages in the same bins as the inner plot. A summary of complete, fragmented, duplicated and missing BUSCO genes in the diptera_odb10 set is shown in the top right. An interactive version of this figure is available at https://blobtoolkit.genomehubs.org/view/idCluTigr1.1/dataset/CAKKTE01/snail.

**Figure 3.**
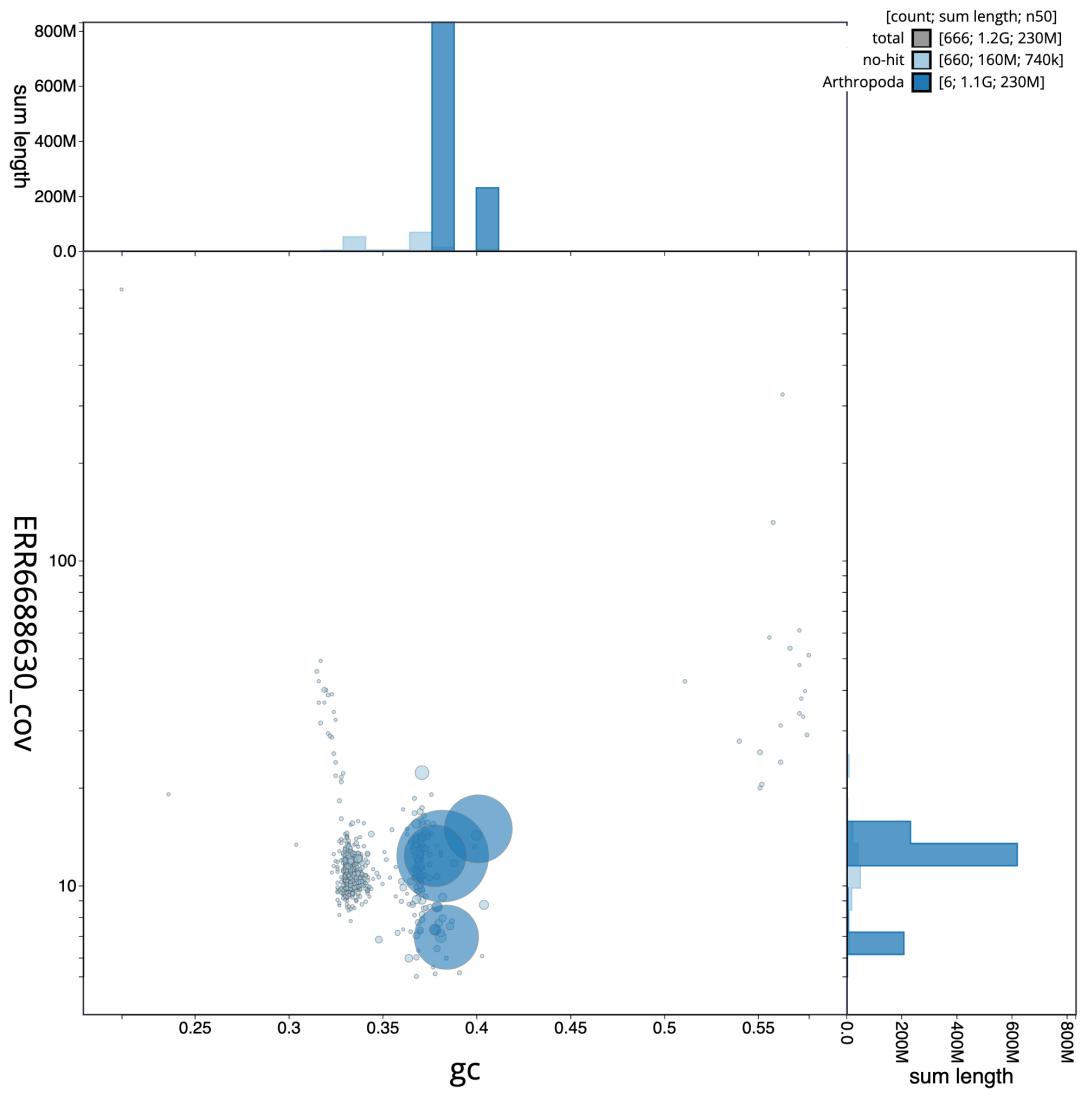
Genome assembly of *Clusia tigrina*, idCluTigr1.2: BlobToolKit GC-coverage plot. Scaffolds are coloured by phylum. Circles are sized in proportion to scaffold length. Histograms show the distribution of scaffold length sum along each axis. An interactive version of this figure is available at https://blobtoolkit.genomehubs.org/view/idCluTigr1.1/dataset/CAKKTE01/blob.

**Figure 4.**
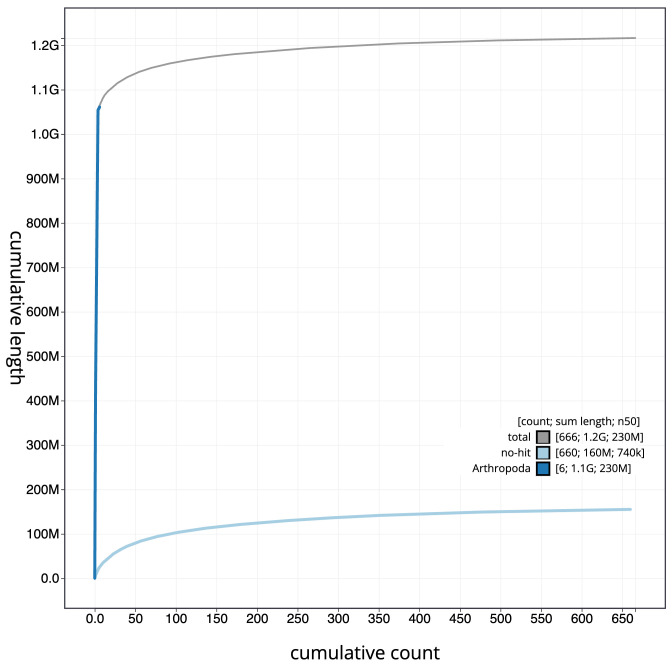
Genome assembly of *Clusia tigrina*, idCluTigr1.2: BlobToolKit cumulative sequence plot. The grey line shows cumulative length for all scaffolds. Coloured lines show cumulative lengths of scaffolds assigned to each phylum using the buscogenes taxrule. An interactive version of this figure is available at https://blobtoolkit.genomehubs.org/view/idCluTigr1.1/dataset/CAKKTE01/cumulative.

**Figure 5.**
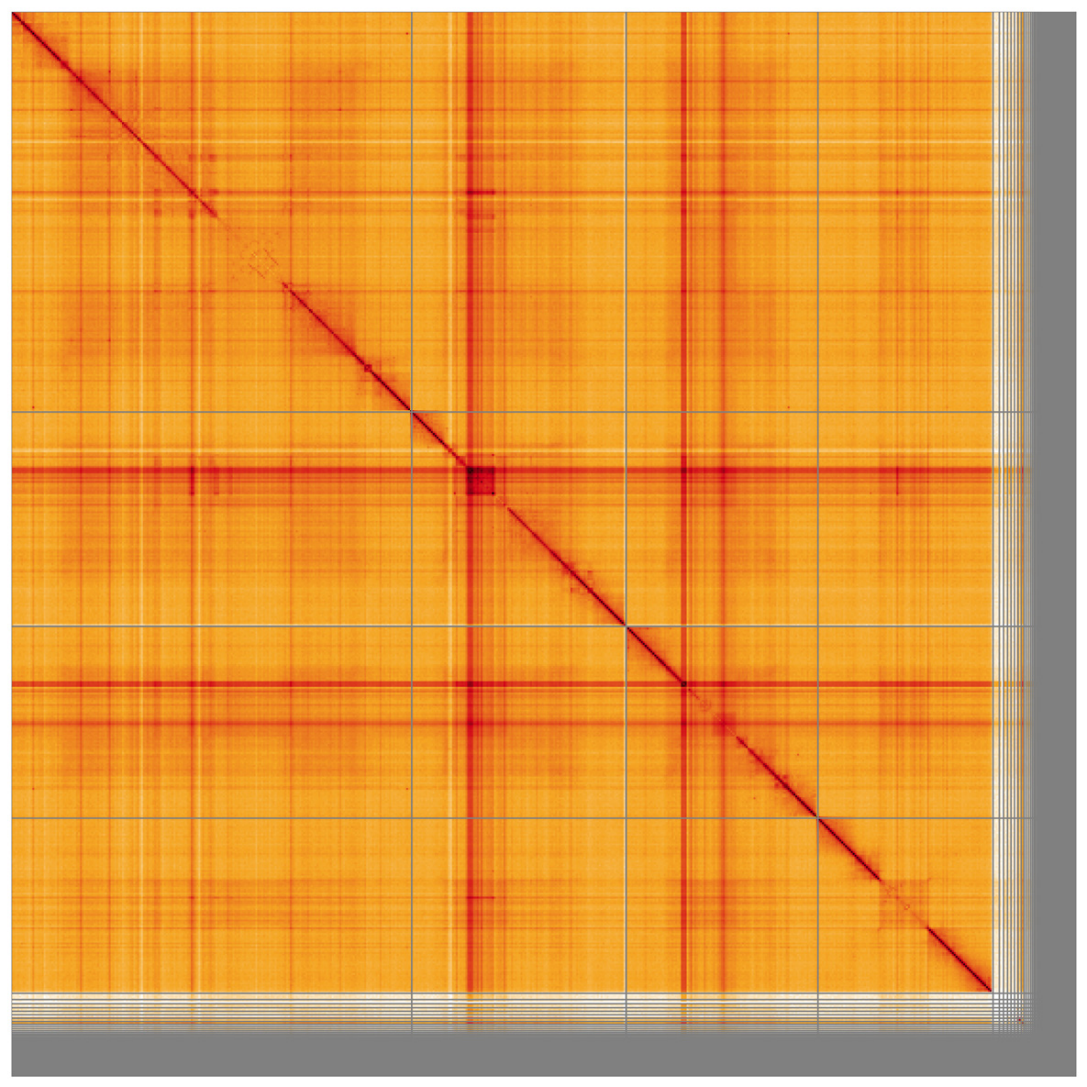
Genome assembly of *Clusia tigrina*, idCluTigr1.2: Hi-C contact map of the idCluTigr1.2 assembly, visualised using HiGlass. Chromosomes are shown in order of size from left to right and top to bottom. An interactive version of this figure may be viewed at https://genome-note-higlass.tol.sanger.ac.uk/l/?d=NWiOFk6uQqSFmLU8Voirqw.

The final assembly has a total length of 1,216.4 Mb in 665 sequence scaffolds with a scaffold N50 of 230.2 Mb ( Table 1). A summary of the assembly statistics is shown in Figure 2, while the distribution of assembly scaffolds on GC proportion and coverage is shown in Figure 3. The cumulative assembly plot in Figure 4 shows curves for subsets of scaffolds assigned to different phyla. Most (92.58%) of the assembly sequence was assigned to 5 chromosomal-level scaffolds, representing 3 autosomes and the X and Y sex chromosomes. Chromosome-scale scaffolds confirmed by the Hi-C data are named in order of size ( Figure 5; Table 2). Chromosome X contains a large region of low confidence from approximately 58.61–118.49 Mb. This block consists of numerous scaffolds with relatively high repeat content where the Hi-C signal is ambiguous in terms of being able to provide a clear order and orientation for the affected scaffolds. In addition, there is a repetitive region of low confidence on Chromosome 2 from approximately 60.32–92.32 Mb, and it was not possible to achieve an accurate order and orientation for the scaffolds in this location. While not fully phased, the assembly deposited is of one haplotype. Contigs corresponding to the second haplotype have also been deposited. The mitochondrial genome was also assembled and can be found as a contig within the multifasta file of the genome submission.

**Table 1.** Genome data for *Clusia tigrina*, idCluTigr1.2.

Project accession data		
Assembly identifier	idCluTigr1.2	
Species	*Clusia tigrina*	
Specimen	idCluTigr1	
NCBI taxonomy ID	576317	
BioProject	PRJEB46850	
BioSample ID	SAMEA7701567	
Isolate information	idCluTigr1, male: whole organism (DNA sequencing) idCluTigr2, female: whole organism (Hi-C scaffolding)	
Assembly metrics *		*Benchmark*
Consensus quality (QV)	51.4	*≥ 50*
*k*-mer completeness	99.96%	*≥ 95%*
BUSCO **	C:97.3%[S:96.0%,D:1.3%], F:0.7%,M:2.0%,n:3,285	*C ≥ 95%*
Percentage of assembly mapped to chromosomes	92.58%	*≥ 95%*
Sex chromosomes	X and Y	*localised homologous pairs*
Organelles	Mitochondrial genome assembled	*complete single alleles*
Raw data accessions		
PacificBiosciences SEQUEL II	ERR6907893, ERR6939254	
10X Genomics Illumina	ERR6688630–ERR6688633	
Hi-C Illumina	ERR6688634	
Genome assembly		
Assembly accession	GCA_920105625.2	
*Accession of alternate haplotype*	GCA_920105815.1	
Span (Mb)	1,216.4	
Number of contigs	1,127	
Contig N50 length (Mb)	5.7	
Number of scaffolds	666	
Scaffold N50 length (Mb)	230.2	
Longest scaffold (Mb)	429.8	

* Assembly metric benchmarks are adapted from column VGP-2020 of “Table 1: Proposed standards and metrics for defining genome assembly quality” from ( Rhie *et al.*, 2021). ** BUSCO scores based on the diptera_odb10 BUSCO set using v5.3.2. C = complete [S = single copy, D = duplicated], F = fragmented, M = missing, n = number of orthologues in comparison. A full set of BUSCO scores is available at https://blobtoolkit.genomehubs.org/view/idCluTigr1.1/dataset/CAKKTE01/busco.

**Table 2.** Chromosomal pseudomolecules in the genome assembly of *Clusia tigrina*, idCluTigr1.

INSDC accession	Chromosome	Length (Mb)	GC%
OV050030.1	1	429.82	38.0
OV050031.1	2	230.18	40.0
OV050033.1	3	187.99	38.0
OV050032.1	X	206.07	38.5
OV050034.1	Y	6.71	37.0
OV050035.2	MT	0.02	21.0

The estimated Quality Value (QV) of the final assembly is 51.4 with *k*-mer completeness of 99.96%, and the assembly has a BUSCO v5.3.2 completeness of 97.3% (single = 96.0%, duplicated = 1.3%), using the diptera_odb10 reference set ( *n* = 3,285).

Metadata for specimens, spectral estimates, sequencing runs, contaminants and pre-curation assembly statistics can be found at https://links.tol.sanger.ac.uk/species/576317.

## Methods

### Sample acquisition and nucleic acid extraction

One male (specimen ID Ox000706, idCluTigr1) and one female (specimen ID Ox000707, idCluTigr2) of *Clusia tigrina* were collected from Wytham Woods, Oxfordshire (biological vice-county Berkshire), UK (latitude 51.76, longitude –1.32) on 2020-07-24. Liam Crowley (University of Oxford) collected and identified the specimens, which were then preserved on dry ice. The male specimen (idCluTigr1) was used for genome sequencing, while the female (idCluTigr2) was used for Hi-C scaffolding.

DNA was extracted at the Tree of Life Laboratory, Wellcome Sanger Institute (WSI). The idCluTigr1 sample was weighed and dissected on dry ice. Tissue from the whole organism was disrupted using a Nippi Powermasher fitted with a BioMasher pestle. High molecular weight (HMW) DNA was extracted using the Qiagen MagAttract HMW DNA extraction kit. Low molecular weight DNA was removed from a 20 ng aliquot of extracted DNA using the 0.8X AMpure XP purification kit prior to 10X Chromium sequencing; a minimum of 50 ng DNA was submitted for 10X sequencing. HMW DNA was sheared into an average fragment size of 12–20 kb in a Megaruptor 3 system with speed setting 30. Sheared DNA was purified by solid-phase reversible immobilisation using AMPure PB beads with a 1.8X ratio of beads to sample to remove the shorter fragments and concentrate the DNA sample. The concentration of the sheared and purified DNA was assessed using a Nanodrop spectrophotometer and Qubit Fluorometer and Qubit dsDNA High Sensitivity Assay kit. Fragment size distribution was evaluated by running the sample on the FemtoPulse system.

### Sequencing

Pacific Biosciences HiFi circular consensus and 10X Genomics read cloud DNA sequencing libraries were constructed according to the manufacturers’ instructions. DNA sequencing was performed by the Scientific Operations core at the WSI on Pacific Biosciences SEQUEL II (HiFi) and Illumina NovaSeq 6000 (10X) instruments. Hi-C data were also generated from whole organism tissue of idCluTigr2 using the Arima2 kit and sequenced on the Illumina NovaSeq 6000 instrument.

### Genome assembly, curation and evaluation

Assembly was carried out with Hifiasm ( Cheng *et al.*, 2021), and haplotypic duplication was identified and removed with purge_dups ( Guan *et al.*, 2020). One round of polishing was performed by aligning 10X Genomics read data to the assembly with Long Ranger ALIGN, calling variants with FreeBayes ( Garrison & Marth, 2012). The assembly was then scaffolded with Hi-C data (Rao *et al.*, 2014) using SALSA2 ( Ghurye *et al.*, 2019). The assembly was checked for contamination and corrected as described previously ( Howe *et al.*, 2021). Manual curation was performed using HiGlass ( Kerpedjiev *et al.*, 2018) and Pretext ( Harry, 2022). The mitochondrial genome was assembled using MitoHiFi ( Uliano-Silva *et al.*, 2023), which runs MitoFinder ( Allio *et al.*, 2020) or MITOS ( Bernt *et al.*, 2013) and uses these annotations to select the final mitochondrial contig and to ensure the general quality of the sequence.

A Hi-C map for the final assembly was produced using bwa-mem2 ( Vasimuddin *et al.*, 2019) in the Cooler file format ( Abdennur & Mirny, 2020). To assess the assembly metrics, the *k*-mer completeness and QV consensus quality values were calculated in Merqury ( Rhie *et al.*, 2020). This work was done using Nextflow ( Di Tommaso *et al.*, 2017) DSL2 pipelines “sanger-tol/readmapping” ( Surana *et al.*, 2023a) and “sanger-tol/genomenote” ( Surana *et al.*, 2023b). The genome was analysed within the BlobToolKit environment ( Challis *et al.*, 2020) and BUSCO scores ( Manni *et al.*, 2021; Simão *et al.*, 2015) were calculated.

Table 3 contains a list of relevant software tool versions and sources.

**Table 3.** Software tools: versions and sources.

Software tool	Version	Source
BlobToolKit	4.1.5	https://github.com/blobtoolkit/blobtoolkit
BUSCO	5.3.2	https://gitlab.com/ezlab/busco
FreeBayes	1.3.1-17-gaa2ace8	https://github.com/freebayes/freebayes
gEVAL	N/A	https://geval.org.uk/
Hifiasm	0.15.3	https://github.com/chhylp123/hifiasm
HiGlass	1.11.6	https://github.com/higlass/higlass
Long Ranger ALIGN	2.2.2	https://support.10xgenomics.com/genome-exome/software/pipelines/latest/advanced/other-pipelines
Merqury	MerquryFK	https://github.com/thegenemyers/MERQURY.FK
MitoHiFi	2	https://github.com/marcelauliano/MitoHiFi
PretextView	0.2	https://github.com/wtsi-hpag/PretextView
purge_dups	1.2.3	https://github.com/dfguan/purge_dups
SALSA	2.2	https://github.com/salsa-rs/salsa
sanger-tol/genomenote	v1.0	https://github.com/sanger-tol/genomenote
sanger-tol/readmapping	1.1.0	https://github.com/sanger-tol/readmapping/tree/1.1.0

### Wellcome Sanger Institute – Legal and Governance

The materials that have contributed to this genome note have been supplied by a Darwin Tree of Life Partner. The submission of materials by a Darwin Tree of Life Partner is subject to the **‘Darwin Tree of Life Project Sampling Code of Practice’**, which can be found in full on the Darwin Tree of Life website here. By agreeing with and signing up to the Sampling Code of Practice, the Darwin Tree of Life Partner agrees they will meet the legal and ethical requirements and standards set out within this document in respect of all samples acquired for, and supplied to, the Darwin Tree of Life Project.

Further, the Wellcome Sanger Institute employs a process whereby due diligence is carried out proportionate to the nature of the materials themselves, and the circumstances under which they have been/are to be collected and provided for use. The purpose of this is to address and mitigate any potential legal and/or ethical implications of receipt and use of the materials as part of the research project, and to ensure that in doing so we align with best practice wherever possible. The overarching areas of consideration are:

• Ethical review of provenance and sourcing of the material

• Legality of collection, transfer and use (national and international) 

Each transfer of samples is further undertaken according to a Research Collaboration Agreement or Material Transfer Agreement entered into by the Darwin Tree of Life Partner, Genome Research Limited (operating as the Wellcome Sanger Institute), and in some circumstances other Darwin Tree of Life collaborators.

## Data Availability

European Nucleotide Archive: *Clusia tigrina*. Accession number PRJEB46850; https://identifiers.org/ena.embl/PRJEB46850. ( Wellcome Sanger Institute, 2021) The genome sequence is released openly for reuse. The *Clusia tigrina* genome sequencing initiative is part of the Darwin Tree of Life (DToL) project. All raw sequence data and the assembly have been deposited in INSDC databases. The genome will be annotated using available RNA-Seq data and presented through the Ensembl pipeline at the European Bioinformatics Institute. Raw data and assembly accession identifiers are reported in Table 1.

## Author information

Members of the University of Oxford and Wytham Woods Genome Acquisition Lab are listed here: https://doi.org/10.5281/zenodo.4789928.

Members of the Darwin Tree of Life Barcoding collective are listed here: https://doi.org/10.5281/zenodo.4893703.

Members of the Wellcome Sanger Institute Tree of Life programme are listed here: https://doi.org/10.5281/zenodo.4783585.

Members of Wellcome Sanger Institute Scientific Operations: DNA Pipelines collective are listed here: https://doi.org/10.5281/zenodo.4790455.

Members of the Tree of Life Core Informatics collective are listed here: https://doi.org/10.5281/zenodo.5013541.

Members of the Darwin Tree of Life Consortium are listed here: https://doi.org/10.5281/zenodo.4783558.
