# Supplementary material for: The genome sequence of a druid fly, Clusia tigrina (Fallén, 1820)
Source: Wellcome Open Res. 2023 Oct 6;8:430. [Version 1] doi: 10.12688/wellcomeopenres.20109.1 (PMC10915360; doi:10.12688/wellcomeopenres.20109.1)
Supplement: Peer review history

# Reviewer report: Reviewer response for version 1

**DOI:** 10.21956/wellcomeopenres.22268.r74654

**Published:** 2024-03-14

**Authors:** Zheng Li

**Affiliations:** 1 The University of Arizona, Tucson, AZ, USA

This paper provides a chromosome-level genome assembly of a druid fly, *Clusia tigrina. *This genome will be a wonderful resource for researchers interested in fly or insect genomics in general. The quality of the genome assembly meets the standard of other insect genomes from the Darwin tree of life project. This manuscript also did a great job as a data note. 

 One thing that can be improved is to provide a citation for karyotyping information. The current inference of chromosome number is based on the Hi-C analysis. Given that the blocks of the chromosome seem ambiguous, it will be stronger to provide additional evidence of the chromosome number.

Are sufficient details of methods and materials provided to allow replication by others?

Yes

Is the rationale for creating the dataset(s) clearly described?

Yes

Are the datasets clearly presented in a useable and accessible format?

Yes

Are the protocols appropriate and is the work technically sound?

Yes

Reviewer Expertise:

insect genomics

I confirm that I have read this submission and believe that I have an appropriate level of expertise to confirm that it is of an acceptable scientific standard.

# Reviewer report: Reviewer response for version 1

**DOI:** 10.21956/wellcomeopenres.22268.r74650

**Published:** 2024-03-14

**Authors:** Daniel Bruzzese

**Affiliations:** 1 Department of epidemiology and Microbial Diseases, Yale University, New Haven, Connecticut, USA

Crowley and Akinmusola have assembled a high quality near-chromosomal 1.2 Gb genome for the druid fly ( *Clusia* *tigrina*) using PacBio, 10X, and Hi-C They then manually curated the assembly and provided thorough quality control to verify the accuracy of their assembly. 

 This is an excellent paper that utilizes the current gold-standard in assembly tools and analysis. and presents a valuable genomic resource.

Are sufficient details of methods and materials provided to allow replication by others?

Yes

Is the rationale for creating the dataset(s) clearly described?

Yes

Are the datasets clearly presented in a useable and accessible format?

Yes

Are the protocols appropriate and is the work technically sound?

Yes

Reviewer Expertise:

Evolutionary biology, symbiosis, and arthropod genomics.

I confirm that I have read this submission and believe that I have an appropriate level of expertise to confirm that it is of an acceptable scientific standard.

# Reviewer report: Reviewer response for version 1

**DOI:** 10.21956/wellcomeopenres.22268.r74651

**Published:** 2024-03-05

**Authors:** Jan Veenstra

**Affiliations:** 1 INCIA, UMR 5287, CNRS, University Bordeaux, Bordeaux, Gironde, France

This is yet another high-quality genome assembly from the Welcome Sanger Institute. It is a very nice illustration of how good today’s technology has gotten with respect to both the physical and digital machinery. This is perhaps best illustrated by having only two authors manuscript describing this genome assembly, as compared to the very long author lists of earlier publications.

 In order to review a genome assembly paper, there are two things to look at, the actual genome assembly, and the manuscript describing it. I spent some time looking for genes I am interested in in this assembly. It is really a good and nice assembly. As to the manuscript itself, I don’t see any missing details.

 The Rao *et al.*, 2014 reference in the text misses the hyperlink.

Are sufficient details of methods and materials provided to allow replication by others?

Yes

Is the rationale for creating the dataset(s) clearly described?

Yes

Are the datasets clearly presented in a useable and accessible format?

Yes

Are the protocols appropriate and is the work technically sound?

Yes

Reviewer Expertise:

Arthropod neuropeptides. Biochemsitry, physiology, molecular biology, evolution.

I confirm that I have read this submission and believe that I have an appropriate level of expertise to confirm that it is of an acceptable scientific standard.

# Reviewer report: Reviewer response for version 1

**DOI:** 10.21956/wellcomeopenres.22268.r74653

**Published:** 2024-03-04

**Authors:** Tomas N. Generalovic

**Affiliations:** 1 Zoology, University of Cambridge, Cambridge, England, UK

This article presents a genome assembly for the species *Clusia tigrine*, a species of which appears to be of primary interest in biodiversity monitoring and morphological analysis. This genome assembly is of high-quality and adds a significant and relatively complete resource to the public domain. Whilst reports on mitochondrial and nuclear markers have been reported for Clusiidae species [1], this appears to be the first whole genome resource available for the Clusiidae. The 1.2 Gb genome of the druid fly is assembled into three autosomes and two sex chromosomes. A typical XY sex determination system is identified. This reference is generated from a male individual and an array of sequencing technology allowing (pseudo-)chromosomal scaffolds to be assembled. This resource will likely be of benefit for studying the evolutionary trajectory of the species and wider genera and appears of particular interest in behavioural investigations but seemingly the most beneficial insights will be to monitor biodiversity. This fly was sampled in the UK (Wytham Woods, Oxford) where it appears to be a rare observation.

 The genome appears to have several scaffolds orientated with low confidence, however, overall, the assembly appears to exceed several of the general benchmarks suggested by [2], with the exception of “Percentage of assembly mapped to chromosomes”. The second (incomplete) haplotype was also only assembled to contig-level with only 540.9 Mb sequence assembled.

 The rationale for this work is clear and the methodological approach is using up-to-date technology. All interactive links appear in working order and the data is available in the public domain as stated.

 Overall, this article is scientifically sound as a data note with only minor comments related to grammar etc. which are provided below:

 Several incidents of values less than 10 being referred to in the numerical form, values <10 e.g. “5” instead of “five”. Advice changes throughout.

 Under the Data availability statement the Wellcome Sanger Institute, 2021 reference is incorrectly placed after the full top.

Are sufficient details of methods and materials provided to allow replication by others?

Yes

Is the rationale for creating the dataset(s) clearly described?

Yes

Are the datasets clearly presented in a useable and accessible format?

Yes

Are the protocols appropriate and is the work technically sound?

Yes

Reviewer Expertise:

Evolutionary Biology

I confirm that I have read this submission and believe that I have an appropriate level of expertise to confirm that it is of an acceptable scientific standard.

## References

[1] : Phylogenetic analysis of the druid flies (Diptera: Schizophora: Clusiidae) based on morphological and molecular data. *Insect Systematics & Evolution* .2010;41(3) : 10.1163/187631210X500628 231-274 10.1163/187631210X500628

[2] : Towards complete and error-free genome assemblies of all vertebrate species. *Nature* .2021;592(7856) : 10.1038/s41586-021-03451-0 737-746 10.1038/s41586-021-03451-0 PMC808166733911273
